# Marine turtles are not fussy nesters: a novel test of small-scale nest site selection using structure from motion beach terrain information

**DOI:** 10.7717/peerj.2770

**Published:** 2017-01-04

**Authors:** Ilana Kelly, Javier X. Leon, Ben L. Gilby, Andrew D. Olds, Thomas A. Schlacher

**Affiliations:** School of Science and Engineering, University of the Sunshine Coast, Maroochydore, Queensland, Australia

**Keywords:** Citizen science, Geo-morphometry, Beach vegetation, Nest attributes, Conservation

## Abstract

**Background:**

Nest selection is widely regarded as a key process determining the fitness of individuals and viability of animal populations. For marine turtles that nest on beaches, this is particularly pivotal as the nesting environment can significantly control reproductive success.**The aim of this study was to identify the environmental attributes of beaches (i.e., morphology, vegetation, urbanisation) that may be associated with successful oviposition in green and loggerhead turtle nests.

**Methods:**

We quantified the proximity of turtle nests (and surrounding beach locations) to urban areas, measured their exposure to artificial light, and used ultra-high resolution (cm-scale) digital surface models derived from Structure-from-Motion (SfM) algorithms, to characterise geomorphic and vegetation features of beaches on the Sunshine Coast, eastern Australia.

**Results:**

At small spatial scales (i.e., <100 m), we found no evidence that turtles selected nest sites based on a particular suite of environmental attributes (i.e., the attributes of nest sites were not consistently different from those of surrounding beach locations). Nest sites were, however, typically characterised by occurring close to vegetation, on parts of the shore where the beach- and dune-face was concave and not highly rugged, and in areas with moderate exposure to artificial light.

**Conclusion:**

This study used a novel empirical approach to identify the attributes of turtle nest sites from a broader ‘envelope’ of environmental nest traits, and is the first step towards optimizing conservation actions to mitigate, at the local scale, present and emerging human impacts on turtle nesting beaches.

## Introduction

Death is now the phoenix’ nest;

And the turtle’s loyal breast

To eternity doth rest,

From: “*The Phoenix and the Turtle*”

by William Shakespeare (Harrison, 1966).

Habitat selection is a universal biological process in which individuals actively identify and inhabit sub-sections of a broader habitat to increase fitness (Fuentes et al., 2010; Morris, 2011; Schlacher, Meager & Nielsen, 2014). Nest-site selection is a key component of habitat selection (Jones, 2001; Morris, 2011; Schlacher, Meager & Nielsen, 2014), with nest position often resulting from trade-offs that are made by adults to maximise their own survivorship and optimise the fitness of their offspring (Nilsson, 1984; Martin & Roper, 1988; Fuentes, Limpus & Hamann, 2011).

Marine turtles are emblematic flagship species in biological conservation, being threatened globally by the cumulative pressures of harvesting, habitat modification, pollution, and climate change (Kamrowski et al., 2014; Roe et al., 2014; Weber et al., 2014; Fuentes et al., 2015; Jeffers & Godley, 2016). Adult female turtles deposit their eggs in shallow nests on the dunes of sandy beaches, and are believed to select nesting locations to minimise predation risk and to optimise reproductive success (Nel, Punt & Hughes, 2013). The attributes of nest sites control the thermal environment for the developing eggs (Booth & Astill, 2001; Fuentes et al., 2009), and also modify both predation risk and access to the ocean for emerging hatchlings (Mousseau & Fox, 1998; Wood, Bjorndal & Ross, 2000; Putman, Bane & Lohmann, 2010a; Limpus & Kamrowski, 2013). Female turtles may also choose nest positions to minimise their energy expenditure and maximise the ease with which they can return to the sea, thereby increasing the probability of inundation for nests that are constructed lower on beaches (Kamel & Mrosovsky, 2004; Pfaller, Limpus & Bjorndal, 2009). Adult marine turtles provide no post-ovipositional care to their offspring, and so cannot modify nest attributes to compensate for poorly selected nest sites, or changes to the environment near the nest that occur after oviposition (Wood, Bjorndal & Ross, 2000). Consequently, the environmental attributes of nest sites play a critical role in determining hatching success, and in modifying the fitness and survivorship of turtle hatchlings (Mitchell, Warner & Janzen, 2013).

Marine turtles are thought to select nest sites according to a hierarchy of environmental factors operating across a range of spatial scales (Table 1) (Wood, Bjorndal & Ross, 2000; Roe, Clune & Paladino, 2013). At regional scales (10s km), the choice of nest position is thought to be largely determined by variations in weather and oceanographic conditions, as well as the natal homing behaviour of individuals (i.e., philopatry) (Putman, Shay & Lohmann, 2010b; Pike, 2013a; Brothers & Lohmann, 2015). At the scale of individual beaches (100s m), it has been hypothesized that nest site selection by females may be influenced by local environmental conditions, including beach morphology (Wood, Bjorndal & Ross, 2000; Cuevas, de los Ángeles Liceaga-Correa & Mariño-Tapia, 2010), dune vegetation (Turkozan, Yamamoto & Yilmaz, 2011), and sediment attributes (i.e., grain size, sand temperature) (Wood, Bjorndal & Ross, 2000; Fuentes et al., 2010). Despite the widely-cited and hypothesized role of these environmental factors in putatively influencing nesting turtles, it is rare for studies to examine the influence of multiple environmental attributes, making robust attribution and inferences about the relative importance of individual factors difficult or impossible. Furthermore, there is high intra- and interspecific variation in observed relationships between nest position and the highly dynamic features of beaches and their surf-zones (Hamann, Limpus & Owens, 2002; Miller, Limpus & Godfrey, 2003; Liles et al., 2015). Thus, there are no universally accepted and robust models to predict how the environmental attributes of nesting beaches determine nest selection by nesting marine turtles (Liles et al., 2015; Santos et al., 2015).

Anthropogenic impacts, foremost coastal urbanization and climate change, are altering the structure and function of sandy beaches at unprecedented scales and intensities (Schlacher et al., 2007; Schoeman, Schlacher & Defeo, 2014; Schlacher et al., 2015; Schlacher et al., 2016), and may have changed the quality of many sandy beaches as a nesting habitat for marine turtles (Mazaris, Matsinos & Pantis, 2009; Pike, 2013a; PCC, 2014). Artificial night-light can alter the behaviour of female turtles that emerge to nest on beaches where the light environment is significantly changed (Salmon & Witherington, 1995; Mazaris, Matsinos & Pantis, 2009). Artificial lights can disorient hatchlings and increase the risk of predation (Limpus & Kamrowski, 2013; Rivas et al., 2015; Thums et al., 2016). Many urban beaches have seawalls and are being artificially nourished with sand, detrimentally affecting nesting female turtles and hatchlings (Brock, Reece & Ehrhart, 2009; Rizkalla & Savage, 2010; Fujisaki & Lamont, 2016).

Many populations of marine turtles are of significant conservation concern, requiring multiple management interventions (Harris et al., 2015). One approach (in the broader conservation toolkit for marine turtles) is to actively manage beach- and dune-scapes to optimize conditions for nesting by protecting areas with favourable nest site attributes. To do this, one first requires empirical data on the features of beaches that are characteristic of turtle nests, and therefore represent locations that are likely to be suitable nesting sites. In this context, the chief aim of this study is to identify the environmental features of nesting beaching that are associated with successful turtle oviposition. To this end, we measured a broad suite of local environmental attributes derived from geo-morphometric techniques based on ultra-high resolution digital surface models and imagery. By applying these novel techniques, we introduce two new geomorphic factors to the study of nest site selection in marine turtle research: terrain ruggedness and beach profile curvature. We then test whether these environmental features of the beach- and dune face are associated with nest sites.

## Methods

### Study area

**Figure 1:**
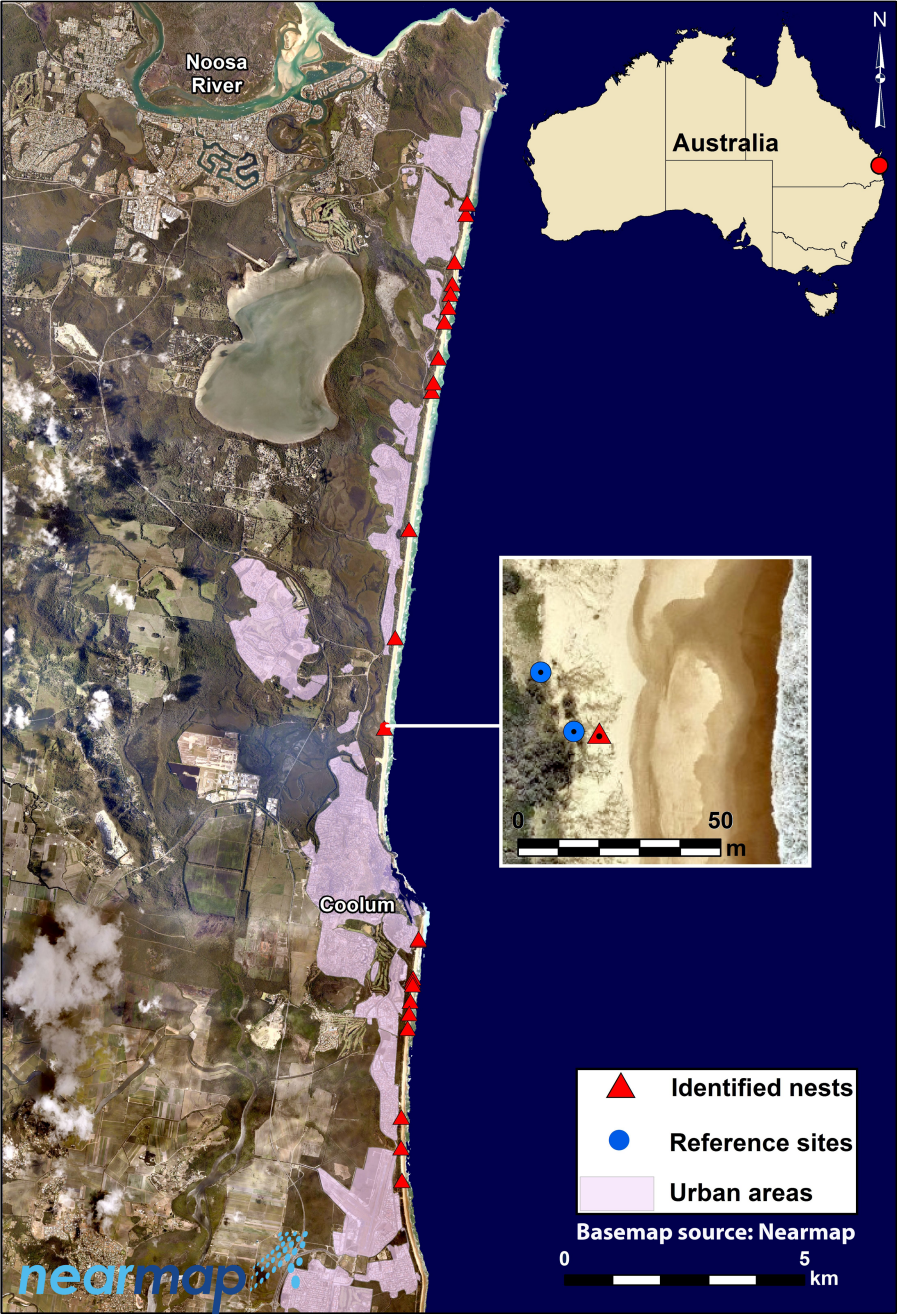
Map of study area on the northern Sunshine Coast in south-east Queensland, Australia. Map shows the location of the 19 nest sites, digitized urban areas, and an insert map illustrating the random ‘reference sites’ measured in this study in the vicinity of a nest site (Map data ©NearMap Pty. Ltd. 2014).

Turtle nest-site attributes were measured along 26 km of exposed ocean beaches on the northern Sunshine Coast in south-east Queensland, Australia (∼26°30′S, 153°6′E) (Fig. 1). These beaches are mostly of the intermediate morphodynamic type (sensu Short & Jackson, 2013), and are micro-tidal (typical range <2 m) with moderate to high waves (significant wave height 0.5–2 m) from prevailing south-easterly winds (Schlacher & Thompson, 2012; Schlacher et al., 2015). Landward development consists mostly of peri-urban to sub-urban private dwellings built on, or behind, a vegetated dune system with an average width of 100–150 m and height of ∼10 m above mean sea level. For the purposes of this study, urban areas are defined as contiguous land cover or land composed of impervious surfaces that include housing, buildings, and other anthropogenic infrastructure such as roads (Huijbers et al., 2015).

**Table 1:** Summary of studies assessing the contribution of different environmental factors to the selection and attributes of marine turtle nests. Specifying species studied, the reported relationship, number of studies the feature was included in and key references.

Environmental factor(s)	Species^a^	Reported general relationship with nest placement	No. studies^b^	Key reference(s)
**Intertidal beach**				
Slope	LH, GT, HB, OR, LB	Highly variable relationship between angle of the beach and nest density or frequency with no consistent pattern. Variability is evident among species and populations, tending to be rookery habitat specific	11 (10)	Garmestani et al. (2000), Wood, Bjorndal & Ross (2000), Fish et al. (2005), Ficetola (2007), Spanier (2009), Cuevas, de los Ángeles Liceaga-Correa & Mariño Tapia (2010), Katselidis et al. (2013b) and Roe, Clune & Paladino (2013)
Width	GT, HB, LH, OR	Highly variable, with evident preferences for both wide and narrow beaches and beach sections. Variability is evident among species and populations, tending to be rookery habitat specific	8 (6)	Kikukawa, Kamezaki & Ota (1999), Garmestani et al. (2000), Mazaris, Matsinos & Margaritoulis (2006), Cuevas, de los Ángeles Liceaga-Correa & Mariño Tapia (2010), Witherington, Hirama & Mosier (2011), Katselidis et al. (2013b) and Barik et al. (2014)
Elevation	LH, HB	Positive correlation with nest density for LH and HB, nesting consistently occurred at a specific elevation.	3 (3)	Horrocks & Scott (1991), Kikukawa, Kamezaki & Ota (1999) and Katselidis et al. (2013a)
Topography	LH, GT	Positive correlation with uneven beach topography for GT, with nest excavation believed to be initiated by the presence of the uneven beach zone above the spring high tide line.	1 (1)	Hays et al. (1995)
Ordinal aspect	LH, HB	Not significant	1 (0)	Garmestani et al. (2000)
**Dune**				
Silhouette	LH	Higher emergences on beach sections where dunes have a distinct and/or higher silhouette.	5 (4)	Camhi (1993), Hays & Speakman (1993); Salmon & Witherington (1995), Mazaris, Matsinos & Margaritoulis (2006) and Witherington, Hirama & Mosier (2011)
Slope	HB, GT	Not significant	1 (0)	Cuevas, de los Ángeles Liceaga-Correa & Mariño Tapia (2010)
**Sediment**				
Grain size	OR, LB, LH, GT	Nest density is positively correlated with medium-sized grains for OR, intermediate size classes for LB, and large particle size classes for LH. LH and LB fewer nests in areas with silty sediment). GT nesting in a range of sediment grain sizes.	5 (4)	Horrocks & Scott (1991), Garmestani et al. (2000), Wood, Bjorndal & Ross (2000), Karavas et al. (2005), Roe, Clune & Paladino (2013) and Barik et al. (2014)
Sorting	LH, OR	Higher nest density in areas with well-sorted sand grains.	3 (3)	Mazaris, Matsinos & Margaritoulis (2006), Chen, Cheng & Hong (2007) and Barik et al. (2014)
Compaction	HB, LH, GT	HB and LH nest density positively correlated with lower sand compaction, with higher rates of nest abandonment in areas of highly compacted sands. GT nest density higher in areas with higher compaction (i.e., 10–30% vegetation cover) compared to opened sand areas, but lower sand compaction compared to vegetated areas > 40% cover	3 (3)	Kikukawa, Kamezaki & Ota (1999), Chen, Cheng & Hong (2007) and Ficetola (2007)
Temperature	LH	The role of temperature in nest selection is unclear. Stoneburner & Richardson (1981) reported that the rapid increase in surface sand temperature along the water-to-dune axis initiated nesting of loggerhead turtles; however, this was later identified as an artefact of their sampling method (refer to Hays et al., 1995; Wood, Bjorndal & Ross, 2000).	4 (1)	Stoneburner & Richardson (1981), Horrocks & Scott (1991), Hays et al. (1995) and Wood, Bjorndal & Ross (2000)
Moisture	LH, GT	Successful nesting attempts in GT associated with higher sand moisture, while unsuccessful nesting attempts in drier sand.	5 (1)	Bustard & Greenham (1968), Garmestani et al. (2000), Wood, Bjorndal & Ross (2000) and Chen, Cheng & Hong (2007)
Salinity	LH, LB	Significant factor only for LB, showing a negative correlation with nest density.	2 (1)	Karavas et al. (2005); Roe, Clune & Paladino (2013)
pH	LH, LB, GT, HB	Highly variable relationship between nesting and pH: positive in LB, negative in HB, no association in GT.	4 (2)	Garmestani et al. (2000), Karavas et al. (2005), Mazaris, Matsinos & Margaritoulis (2006) and Barik et al. (2014)
Organic content	LB, LH	Not significant	4 (0)	Horrocks & Scott (1991), Karavas et al. (2005), Mazaris, Matsinos & Margaritoulis (2006) and Roe, Clune & Paladino (2013)
Calcium carbonate content	LH	Nesting density positively correlated with low calcium carbonate content.	1 (1)	Garmestani et al. (2000)
Rock cover	HB	Nesting positively correlated with low rock cover and higher nest abandonments in areas with higher rock cover.	1 (1)	Ficetola (2007)
**Vegetation**				
Cover	LH, GT, HB, LB, OR	Significant factor, however, the nature of the relationship varies greatly among populations. LH and OR population’s preferred bare sand areas, generally aborting nesting attempts in vegetation cover. A single study identified successful nesting in vegetation for LH but at a lower density to open sand nesting. LB nesting density is higher on bare sand or negligible vegetation cover. GT nest density is higher in the vegetated zones (particularly in 10–30% vegetation cover), nesting still occurs on the un-vegetated zone of beach but to a lesser degree. HB nesting density highest in dense shrub coverage.	11 (11)	Hays & Speakman (1993), Hays et al. (1995), Kikukawa, Kamezaki & Ota (1999), Kamel & Mrosovsky (2004), Karavas et al. (2005), Mazaris, Matsinos & Margaritoulis (2006), Chen, Cheng & Hong (2007), Ficetola (2007), Serafini, Lopez & Da Rocha (2009), Turkozan, Yamamoto & Yilmaz (2011) and Hart et al. (2014)
Canopy cover (%)	HB	HB population in the West Indies selected a variety of canopy cover, with significant individual repeatability in the percentage of canopy cover used. While, HBs of El Salvador and Nicaragua had strong population preferences for abundant over story vegetation cover (84.1% and 92.5%, respectively)	3 (3)	Kamel & Mrosovsky (2005), Kamel & Mrosovsky (2006) and Liles et al. (2015)
Species composition	LH, HB	LH did not nest in vegetated zones of the beach, which were dominated by woody shrubs and trees, though some nesting (10/180 nests) occurred in areas of low-lying vegetation with rhizomes. HB show individual preferences for vegetation coverage of low lying grass and tall woody vegetation	5 (4)	Garmestani et al. (2000), Kamel & Mrosovsky (2005), Karavas et al. (2005) and Kamel & Mrosovsky (2006)

**Notes.**

a LH, Loggerhead; GT, Green turtle; LB, Leatherback; HB, Hawksbill; OR, Olive ridley.

b Number of studies that report statistically significant relationships between nesting density/frequency and a particular environmental factor is given in brackets.

In this region, nesting occurs primarily from early November to mid-February mainly by loggerhead turtles (*Caretta caretta*) and, to a lesser extent, by green turtles (*Chelonia mydas*) (Limpus, 2008; Limpus & Fien, 2008). The number of loggerhead turtle nests in the region varies from 4 to 30 confirmed nest locations per season (Coolum Coast Care & I Kelly, pers. comm., 2014). Overall, the number of nesting loggerhead females on the northern Sunshine Coast represents approximately 0.15–1.11% of the Australian east coast population (approximately 2,700 individuals; Limpus & Limpus, 2003; Kamrowski et al., 2014). Green turtle nests are less common, with fewer than 5 nests recorded each nesting season on this particular stretch of coast. Whilst the beaches of the Sunshine Coast currently only support a comparatively small part of the nesting population of marine turtles on the East Coast of Australia, the region may be at the leading edge of predicted species range shifts associated with climate change and hence become more important for nesting turtles in the future.

During this study, nests were located by the tracks made by female turtles when crossing the beach and dunes. Citizen scientists from a local community group (Coolum Coast Care) monitored all beaches daily from early November 2014 to late February 2015: we are confident that all nests were successfully located along the northern Sunshine Coast beaches within this period.

Within two hours following detection of tracks by volunteers, we measured a suite of variables at turtle nests in which eggs were successfully deposited, and at two random reference sites within a 50 m radius of each nest. We determined the position of random reference sites using a random number generator in ArcGIS 10.2 (ESRI, 2013), but stipulated that they must occur at least 5 m from a known nest site. These reference sites at which turtles did not attempt to construct nests, therefore, constitute a suite of locations where environmental conditions are theoretically appropriate for turtle nesting, but where no turtle nested during our study.

### Terrain data collection

Ultra-high spatial resolution (cm-scale) image orthomosaics and digital surface models (DSMs) of the beach and dune areas surrounding observed nests (∼50 m) were derived by close-range photogrammetry and Structure from Motion (SfM) algorithms. SfM semi-automated photogrammetric technique is useful for obtaining ultra-high resolution datasets and 3D information from 2D images (Leon et al., 2015). The technique is now used in a diverse range of applications, including quantifying geomorphic features on sandy beaches (Mancini et al., 2013; Chikhradze et al., 2015). SfM works by using a set of overlapping 2D digital images and matches single features in multiple images to reconstruct 3D geometry (Westoby et al., 2012; Mancini et al., 2013).

Images were obtained with a digital camera (Canon PowerShot D30, 12.1 megapixels, ∼AU$300), programmed to take images every second using the Canon Hack Development Kit (http://chdk.wikia.com/wiki/CHDK). The camera was mounted on an 8 m pole and tilted obliquely at a 30° angle to reduce systematic broad-scale errors (i.e., “doming” effect) in the topographic reconstructions (James & Robson, 2014). The camera was carried by the first author walking a series of parallel, overlapping transects up and down the width of the beach, within a quadrat set at ∼50 m by the length of the beaches width (max area covered 250 m^2^) and encompassing the nest itself. Accurate SfM requires at least 70% overlap between images and at least three consecutive images within which a given feature (i.e., key point) is visible (Westoby et al., 2012). Each image produced a footprint of 10 × 7 m at a height of 8 m, so each image and every transect line were separated by a maximum of 3 m. Five ground-control points (GCPs; discs marked with two 20 cm scale bars) were placed at the corners and middle of the surveyed quadrat and their location recorded using a handheld Garmin eTrex 10 GPS (horizontal accuracy < 2 m). These ground-control points were used to geo-reference the cameras’ absolute position (i.e., object-space coordinates). Seven additional GCPs were randomly distributed across the quadrat and were used for precise scaling and optimization (relative position) of the 3D model and image orthomosaic.

Agisoft PhotoScan Professional edition v1.1 software was used to create the DSMs and image orthophoto mosaics based on SfM algorithms. Processing involved four main steps: (1) image triangulation, (2) optimization, (3) dense surface reconstruction, and (4) orthophoto generation. The products were georeferenced to GDA94 MGA56 coordinate systems and the resulting orthophoto mosaics and the DSMs were exported with a 1 cm and a 10 cm spatial resolution, respectively.

For comparison purposes, a detailed validation was undertaken at one of the nest locations based on 32 independent, randomly placed ground-control points (GDA94 MGA 56 horizontal coordinate system, AHD vertical datum) surveyed across the quadrat boundary using a high-precision Global Navigation Satellite System (CHC X91+ system with nominal accuracy of 10 mm and 15 mm in horizontal and vertical positions, respectively). The models have a high precision with an average relative accuracy of 0.01 m (horizontal and vertical) as calculated from the used markers and scales. Only one of the models was validated independently for absolute accuracy using a high-precision real time differential GPS. The results showed an average elevation error (absolute accuracy) of 0.22 m from the Australian Height Datum.

### Nest site attributes

Beach geomorphic features were extracted from the SfM-derived DSMs. Terrain analysis was performed using SAGA GIS 2.0 (Cimmery, 2010) to obtain profile curvature (Romstad & Etzelmüller, 2012) and terrain ruggedness (Sappington, Longshore & Thompson, 2007). In total, we measured 11 attributes for each nest and reference site; a full description of all metrics used to define nest attributes is given in Table 2.

**Table 2:** List of the environmental and anthropogenic features and nest attributes measured in this study.

Measured features and attributes	Units	Description	Reference
**Environmental features**			
Slope of subaerial beach and dune face	Degrees	Subaerial beach slope was measured between the observed high-water line and the dune toe perpendicular to the nest sites. Dune slope was measured between the dune toe and the dune crest perpendicular to the nest sites. All distance and elevation were measured using ArcGIS10.2.	Zevenbergen & Thorne (1987), ESRI (2013)
Beach profile curvature	Radians per m	Curvature is the second derivative of elevation (i.e., the slope of the slope) calculated using the SfM-DEM. Profile curvature is the curvature intersecting with the plane defined by the *Z*-axis and maximum gradient direction. Positive values describe convex profile curvature; negative values concave profile and zero values flat surface. Calculated using SAGA GIS 2.1 as implemented in the Morphometry Features tool using a 5 ×5 m window.	Wood, Bjorndal & Ross (2000); Romstad & Etzelmüller (2012)
Terrain ruggedness	Standardised index	Terrain ruggedness was calculated using the vector ruggedness measure (VRM), a parameter that minimizes correlation with slope, based on the SfM-DEM. The dimensionless ruggedness number ranges from 0 (flat) to 1 (most rugged). Calculated in SAGA GIS as implemented in the Morphometry Features tool using a 5 ×5 m window.	Sappington, Longshore & Thompson (2007)
Width of the Subaerial beach and dune	m	The subaerial beach was defined between the observed high-water line and the dune toe perpendicular to nest sites. The dune face was defined between the dune toe and dune crest perpendicular to nest sites. Measured using ArcGIS 10.2	ESRI (2013)
Distance of nest from dense vegetation	m	The Euclidean distance from the nearest digitized dense vegetation cover to the nest as implemented in ArcGIS 10.2.	Hunt et al. (2005); ESRI (2013)
Distance of nest from sparse vegetation	m	The Euclidean distance from the nearest digitized sparse vegetation cover to the nest as implemented in ArcGIS 10.2.	Hunt et al. (2005); ESRI (2013)
**Anthropogenic features**			
Distance to urban areas	m	Euclidean distance from nearest urban area (i.e., contiguous land cover/land composed of relatively dense coverage of impervious surfaces that include housing and other anthropogenic infrastructure) as implemented in ArcGIS 10.2.	ESRI (2013)
Exposure to artificial light	(mcd m^2^)	Manual light measurements using a hand-held night sky brightness photometer, Unihedron Sky Quality Meter-L. Light was measured as magnitudes per square arcsecond (mag/arcsec^2^) and converted to milicandelea per square meter (mcd m^2^).	Cinzano & Falchi (2014)

SfM-derived image orthophoto mosaics were used to manually digitize beach and dune geomorphic features including the dune crest, dune toe, and the observed high-water line (proxy for high tide waterline; Boak & Turner, 2005). The subaerial beach width and slope were measured between the observed high-water line and the dune toe perpendicular to the nest sites. Dune slope and width were measured between the dune toe and the dune crest perpendicular to the nest sites. All distance and elevation were measured using ArcGIS10.2.

Proximity analysis was used to calculate straight-line (Euclidean) distances between each nest and random points and the nearest urban area. These areas were manually digitized from a 2012 SPOT 5-satellite imagery (2.5 m spatial resolution) (See Fig. 1). Distance to vegetation cover on the dune and beach was also calculated in this way. Sparse and dense vegetation coverage was classified using a semi-automatic approach based on the SfM-derived image orthomosaics. Imagery consisted of only visible bands (i.e., red, green, blue), so the normalized green-red difference index (NGRDI) (Hunt et al., 2005) was preferred over conventional indices that require the near-infrared band. Vegetation cover was first classified by manually setting a threshold for the NRGDI images. Vegetation density was then determined by counting how many vegetation-classified pixels were located within 0.5 m^2^ “virtual” quadrats, derived using the Block Statistics tool in ArcGIS 10.2. Finally, vegetation density was classified into sparse (<50%) and dense (>50%) vegetation cover classes.

To identify whether nests and reference sites were exposed to artificial light sources, light measurements were taken with a hand-held night sky brightness photometer (Unihedron Sky Quality Meter-L.) This instrument responds to light with wavelengths in the range of 320–1050 nm, which covers the range marine turtles are known to respond to (i.e., 350–700 nm; Kamrowski et al., 2012). Light was measured as magnitudes per square arcsecond (mag/arcsec^2^) and converted to milicandelea per square meter (mcd/m^2^). This instrument was calibrated using a NIST-traceable light meter with an absolute precision of ±0.10 mag/arcsec^2^. Measurements were recorded at each nest and reference site facing towards the back of the dune. All measurements were taken at ‘turtle-height’ by crouching down and taking a reading about 5–10-cm above the sand surface at the uprush limit of the swash. A beach location, which had no visible sources of artificial light during a new moon period, had a light reading of ∼0.2–4 mcd m^2^ whereas a beach with visible artificial light had a reading of ∼7–14 mcd m^2^.

### Data analyses

Our analyses tested three complementary questions: (1) What environmental conditions are typical of turtle nests; (2) Do the environmental characteristics of turtle nests differ from attributes of the broader dune-face and beach-face; and (3) Which parts of dunes and the upper beach are substantially different (i.e., highly distinct) in their environmental characteristics from nesting locations, and thus probably not suitable for turtle nesting in the future?

To characterise what constitutes the set of ‘typical’ nest site traits (Question 1), we used the similarity percentage (SIMPER) procedure in Primer 6.1.13 (Clarke, 1993) with normalised untransformed data to identify nest attributes that contribute most to the average similarity within the group of nest sites. To test whether nest sites have distinct environmental attributes relative to their nearby (<50 m) surroundings (Question 2), we used a permutational multivariate analysis of variance (PERMANOVA) calculated on a Euclidean distance resemblance matrix (Anderson, Gorley & Clarke, 2008) to contrast features of nest sites with random locations. PERMANOVA was complemented by PERMDISP (homogeneity of dispersions procedure) to test whether nests were more or less variable in multivariate environmental space than the set of reference sites (Anderson, Gorley & Clarke, 2008). Finally, to define areas that would–based on measured attributes of actual nest locations–presumably have a lower probability of successful nesting attempts (Question 3) we used group-average clustering based on Euclidean distances over the full set of environmental variables. Similarities between actual nests, random points within the mean centroid distance of nests, and ‘atypical’ locations were visualised with canonical analysis of principal coordinates (CAP) (Anderson & Willis, 2003). Given our restriction on random sample points being within a 50 m radius of nest sites, all analyses comparing nest-site and random-point attributes are considered to apply to primarily to local scales.

Due to the very low number of green turtle nests (*n* = 2) and diagnostic checks (PERMANOVA), not indicating substantial differences in nest attributes between species, we pooled loggerhead (*n* = 19) and green turtle nests for statistical analyses.

## Results

Environmental factors that explained most of the internal similarity within the group of successfully constructed nest sites were: (1) illuminance; (2) the height of the dune crest; and (3) beach slope (Table 3). Thus, turtles constructed nests on parts of the beach with illuminance values of 2.40–6.03 mcd m^−2^ (95% confidence interval), seawards of dunes that were 5.4–7.3 m high, and landwards of beaches that sloped between 3.5 and 6.7° (Table 3).

**Table 3:** SIMPER (Similarity Percentage) summary statistics. Summary statistics of environmental attributes for observed turtle nest sites. SIMPER (Similarity Percentage) was based on a normalised untransformed data including all listed environmental variables.

			SIMPER		
Variable	Mean (95% CI interval)	Range	Mean squared distance	Sq. dist./SD	Contribution %
Distance to sparse vegetation (m)	1.42 (0.8–1.87)	0–3.52	0.06	0.46	0.63
Terrain ruggedness	0.03 (0.01–0.04)	0–0.10	0.13	0.36	1.36
Distance to dense vegetation (m)	6.12 (2.73–9.51)	1.04–12.74	0.28	0.53	3.03
Profile curvature (rad/m)	−0.01 (−0.04–0.01)	−0.11	0.41	0.44	4.43
Distance to urban land use (m)	178 (104–198)	56–568	0.86	0.35	9.21
Dune slope (deg)	15.76 (10.69–19.19)	3.72–28.75	0.95	0.48	10.21
Dune width (m)	12.18 (7.01–15.93)	2.57–30.97	1.04	0.41	11.17
Beach width (m)	19.34 (12.29–22.96)	2–50.3	1.07	0.41	11.44
Beach slope (deg)	5.16 (3.49–6.71)	−0.67–10.15	1.36	0.47	14.58
Dune crest height (m)	6.43 (5.38–7.34)	3.28–10.54	1.43	0.43	15.36
Illuminance (mcd m^−2^)	4.26 (2.49–6.03)	0.26–13.72	1.74	0.45	18.58

**Figure 2:**
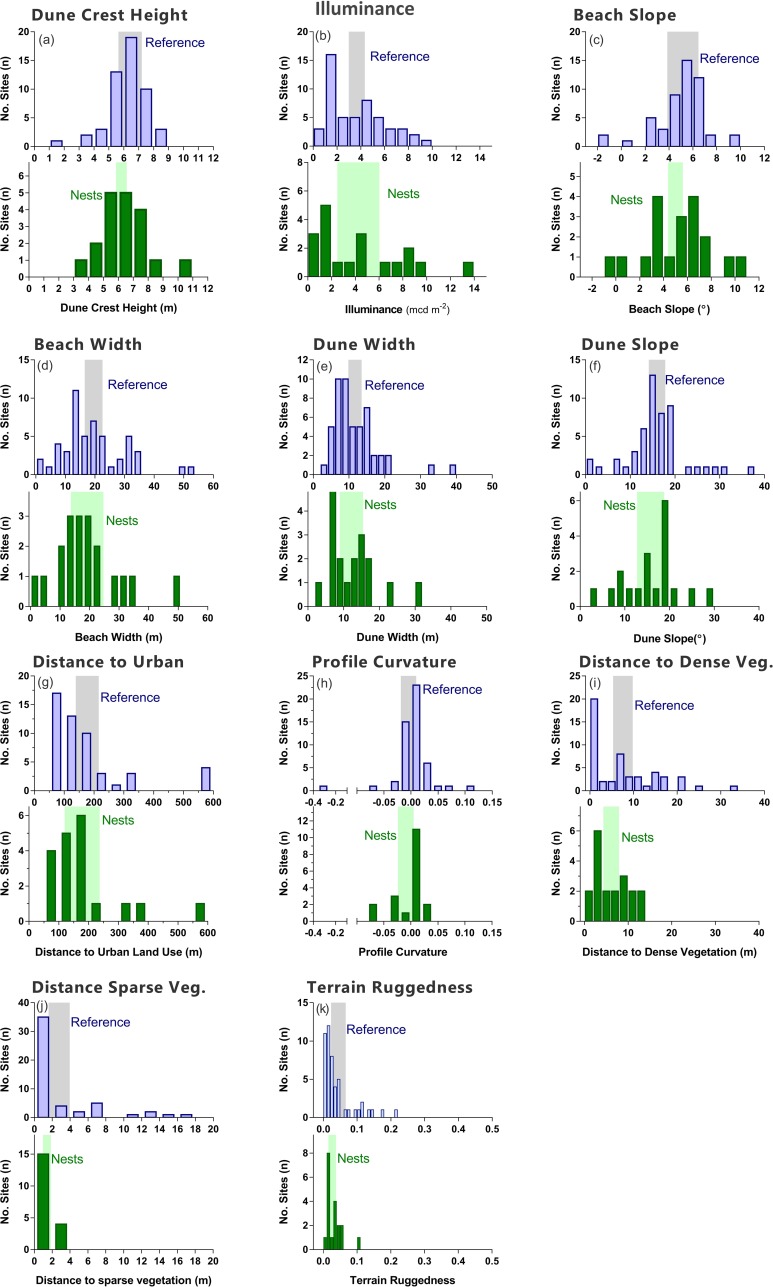
Histograms. Comparison of environmental features between nests (green) and reference sites within 50 m of nests (blue). Shaded areas are 95% confidence intervals.

At small spatial scales (i.e., <100 m), we found no evidence that turtle nests occupy a distinct subset of the broader multi-dimensional geomorphic and vegetation niche present on the upper beach and the frontal dunes (i.e., nest sites did not differ significantly from reference sites) (Table 4, Fig. 2, PERMANOVA *P* = 0.931). Based on mean values of the environmental attributes of nest sites and reference sites, turtles dug nests on parts of the shore where the beach- and dune-face tended to be more concave and less rugged (Fig. 2). Nests were also located closer to vegetation, and in area with slightly higher illumination compared to reference sites (Fig. 2). Mean values for all remaining variables were indistinguishable between nests and reference sites (Table 4).

**Table 4:** Comparison of environmental features between actual nests sites of marine turtles and the full set of random locations sampled within a 50-m radius of each nest site.

Variable	Nests	Reference	Nests	Reference	SIMPER			PERMANOVA	
	Mean (95% Confidence interval)	Mean (95% Confidence interval)	Median (Interquartile range)	Median (Interquartile range)	Average squared distance	Sq. dist./SD	Contrib. %	*F*	*P*
Illuminance (mcd m^2^)	4.26 (2.50–6.03)	3.67 (2.99–4.34)	3.48 (1.64–7.07)	3.14 (1.56–5.31)	2.42	0.66	11.69	0.63	0.46
Dune crest height (m)	6.43 (5.64–7.21)	6.21 (5.85–6.56)	6.33 (5.38–7.34)	6.16 (5.61–7.01)	2.22	0.61	10.75	0.36	0.57
Beach slope (deg)	5.16 (3.85–6.47)	4.99 (4.37–5.61)	5.63 (3.49–6.71)	5.28 (4.19–6.13)	2.17	0.66	10.47	0.07	0.79
Beach width (m)	19.34 (13.96–24.72)	19.56 (16.53–22.59)	17.70 (12.29–22.96)	17.56 (12.7–24.91)	1.99	0.62	9.61	0.01	0.93
Dune width (m)	12.18 (8.93–15.43)	11.67 (9.81–13.53)	10.64 (7.01–15.93)	9.82 (7.6–14.62)	1.98	0.53	9.56	0.08	0.79
Dune slope (deg)	15.76 (12.72–18.79)	15.99 (14.14–17.83)	16.07 (10.69–19.19)	15.90 (12.83–18.5)	1.92	0.62	9.28	0.02	0.90
Distance to urban land use (m)	178 (120–237)	177 (139–215)	151 (104–198)	142 (90–192.8)	1.86	0.52	9.01	0.00	0.97
Profile curvature (rad/m)	−0.0094 (−0.0239–0.0050)	−0.0047 (−0.0191–0.0098)	0 (−0.04–0.01)	0 (−0.01–0.01)	1.61	0.25	7.77	0.17	0.71
Distance to dense vegetation (m)	6.12 (4.31–7.94)	7.56 (5.32–9.80)	4.91 (2.73–9.51)	6.51 (0.2–13.45)	1.55	0.59	7.50	0.57	0.45
Distance to sparse vegetation (m)	1.42 (0.99–1.85)	2.78 (1.60–3.97)	1.35 (0.80–1.87)	0.89 (0.14–3.51)	1.49	0.41	7.19	1.93	0.18
Terrain ruggedness	0.026 (0.015–0.038)	0.044 (0.0227–0.0660)	0.02 (0.01–0.04)	0.02 (0.01–0.04)	1.48	0.24	7.15	0.98	0.36

The illuminance at the beach was the strongest environmental attribute contributing to both the internal similarity within the group of observed nest sites (Table 3), and to the difference between nests and the randomly selected reference sites (Table 4). The mean and median illuminance was slightly higher at nest sites (mean_nest_/mean_reference_ = 1.16; median_nest_/median_reference_ = 1.11), but it must be stressed that these differences are very small, statistically not significant, and show considerable overlap between nest and reference sites (Table 4). Likewise, there was no evidence that selected nest sites were consistently located on substantially darker parts of the beach when analysed at the local (<100 m) scale.

Variability in environmental features was slightly greater at reference sites than at nest sites (PERMDISP *P* = 0.36; distance to centroid: nests = 2.70 ± 0.25 se; reference = 3.06 ± 0.21 se; Table 5). Variation in distance to vegetation (dense and sparse cover) was significantly (*P* < 0.05) greater at reference sites than nest sites (Table 5). Nests were positioned at sites that were slightly, but not significantly, more variable in beach illuminance than reference sites (i.e., illuminance range = 0.26–13.72 mcd m^2^; *P* = 0.07; Table 5).

**Table 5:** Test of the multivariate homogeneity variance in environmental features between turtle nest sites and reference sites. Summary of PERMDISP, testing the multivariate homogeneity of variance in environmental features between turtle nest sites and reference sites within 50 m of nests.

Variable	PERMDISP (*df* 1, 68)		Distance from centroid nests		Distance from centroid reference	
	*F*	*P*	Mean	(se)	Mean	(se)
Distance to dense vegetation (m)	7.84	0.01	3.24	(0.40)	6.35	(0.66)
Distance to sparse vegetation (m)	13.46	0.02	0.69	(0.13)	3.09	(0.40)
Illuminance (mcd m^2^)	4.47	0.07	1.04	(0.18)	0.74	(0.07)
Terrain ruggedness	3.99	0.20	0.02	(<0.01)	0.05	(0.01)
Dune crest height (m)	1.52	0.22	1.22	(0.24)	0.93	(0.12)
Beach slope (deg)	1.71	0.24	2.11	(0.38)	1.57	(0.21)
Dune slope (deg)	0.18	0.69	4.87	(0.87)	4.35	(0.68)
Distance to urban land use (m)	0.18	0.77	80.37	(20.64)	91.55	(14.01)
Dune width (m)	0.13	0.77	5.18	(0.95)	4.74	(0.64)
Beach width (m)	0.07	0.83	7.77	(1.79)	8.28	(0.95)
Profile curvature (red/m)	0.03	0.89	0.023	(0.004)	0.021	(0.007)

**Figure 3:**
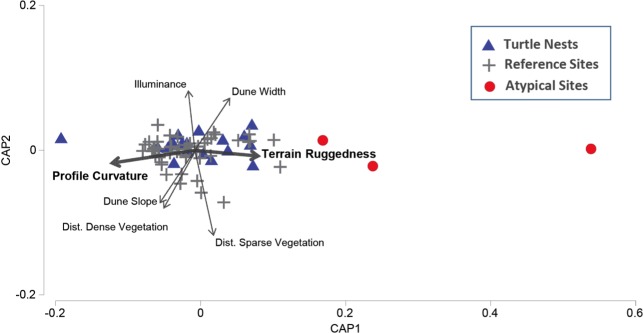
Canonical Analysis of Principle Coordinates (CAP) ordination. Illustrating patterns of similarity in the environmental features of nest sites (green circles), reference sites with similar environmental traits (grey crosses), and those characterized by environmental features that were ‘atypical’ of nest sites (*P* < 0.001) (red stars). Vector width is scaled to the level of correlation (Pearson) with the primary CAP axis.

**Figure 4:**
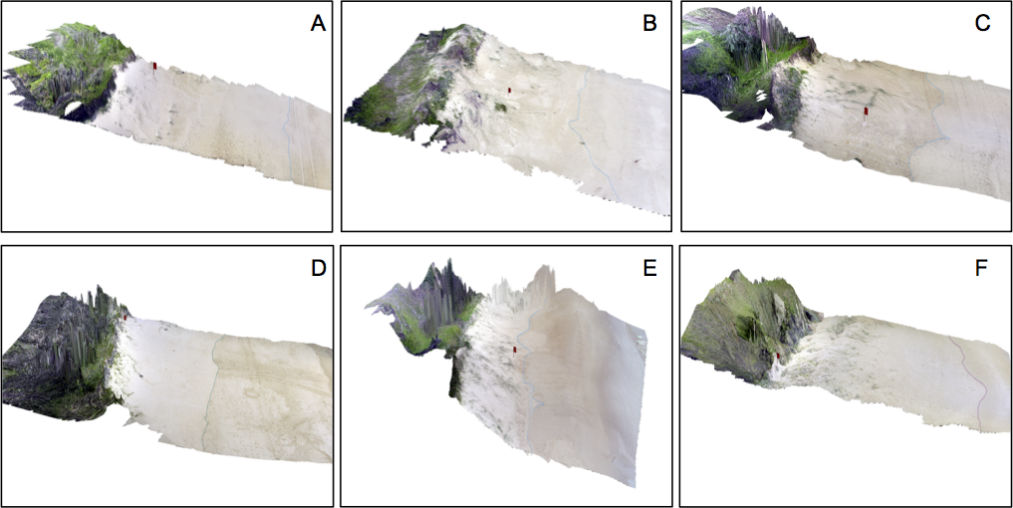
3D perspective of typical and atypical nesting beaches. Typical (A–C) and atypical (D–F) nesting beaches based on CAP analysis with overlaid site locations (red flag) and digitised waterline (blue). Horizontal scale varies with perspective and vertical exaggeration is 1.5×.

Three sites were identified to be distinctly different (i.e., ‘atypical’) to both nest sites and nearby reference sites (Fig. 3). Two ‘atypical’ sites were situated in dense plant cover and in areas characterised by highly rugged terrain (max terrain ruggedness 0.47), one of which was also positioned in a highly concave section of the dune face (profile curvature −0.324). An additional site was located on a very narrow and flat section of the beach (<3 m wide, slope <  0.15°), backed by a low and wide dune (crest height <  2 m, width 40 m) that had a flat frontal face (slope <  0.65°, profile curvature 0.007; Fig. 4).

## Discussion

Recent improvements in the availability and application of remotely sensed data, coupled with new tools for geospatial analysis, provide novel insight into marine turtle nesting patterns and can assist with mapping putative anthropogenic threats to nesting turtles, such as artificial night light (Mazor et al., 2013) and the predicted consequences of seal-level rise (Fish et al., 2005; Fish et al., 2008). We used a combination of close-range photogrammetry and spatial analyses to create ultra-high resolution (cm-scale) terrain and imagery data just hours after turtles had nested. Our study introduces two new geomorphic features to the literature on marine turtle nesting–terrain ruggedness and profile curvature–and supports the importance of some variables (e.g., vegetation), whilst not finding support for others (e.g., urbanisation and illuminance). Nest sites were characterised by occurring close to vegetation, on parts of the shore where the beach- and dune-face was concave and not particularly rugged, and in areas with moderate exposure to artificial light. The environmental attributes of nest sites did, however, not differ significantly from those of surrounding beaches. This finding runs counter to our hypothesis and suggests that turtles might not select nest sites consistently at local scales (<100 m), or that attributes characterising ‘good’ nest sites for turtles were not measured by us. It is also possible that nest site selection may be weaker at or near the range edge of marine turtle nesting which is where our study beaches are located. In addition, we cannot exclude the possibility of multiple nesting by the same female.

The cover of vegetation on dunes was a characteristic of successful nest sites, with most nesting (>90%) occurring in close proximity to vegetation (i.e., within 3.5 m of sparse, and with 13 m of dense vegetation). These findings concur with those of previous studies that report high nesting densities on open sand close to vegetation (Karavas et al., 2005; Chen, Cheng & Hong, 2007; Serafini, Lopez & Da Rocha, 2009; Turkozan, Yamamoto & Yilmaz, 2011; Hart et al., 2014; Katselidis et al., 2013b). The rate of nesting abandonment is, however, also generally highest in vegetated areas (Hays & Speakman, 1993; Karavas et al., 2005). In the study region, dune vegetation is dominated by spinifex grass (*Spinifex longifolius*), which has soft shallow roots that differ fundamentally from the tougher root systems of dune plants reported from other beaches (Hays & Speakman, 1993). Soft-rooted grasses bind sand grains, which helps provide suitable moisture and compactness levels for the construction of egg chambers (Chen, Cheng & Hong, 2007) and nest incubation (Hart et al., 2014). Marine turtles nest successfully near vegetation on numerous beaches, but the roots of dense vegetation also obstruct nest excavation, and so there is likely to be a threshold of vegetation cover above which nesting becomes futile (Chen, Cheng & Hong, 2007). On the beaches we studied, dense vegetation occurs mostly near the crest of dunes, and so we cannot exclude the possibility that vegetation effects on nesting turtles may be confounded with elevation effects.

The profile curvature and terrain ruggedness of beaches differed between nest sites and area of the surrounding beaches that were classified as ‘atypical’ of turtle nests. The relative concave profile of beaches surrounding nest sites may be associated with the proximity of dunes (i.e., beach profiles might dip prior to the dune toe). Alternatively, nests may be placed between embryo dunes near the foot of larger foredunes. Green turtles initiate nest excavation on parts of beaches where the terrain is rugged or uneven, whereas smoother beach profiles are linked to greater rates of nest abandonment (Hays et al., 1995). In our study, turtles constructed nests on sections of beaches with moderately rugged terrain; these values were, however, not significantly different to reference points on surrounding beach locations.

We found no significant effect of urban development (indexed by land conversion) on the location of turtle nests. Beaches in our study area were, however, less urbanized than those examined in other studies that report negative impacts of coastal development on turtle nesting (Weishampel et al., 2003; Kaska et al., 2010; Roe, Clune & Paladino, 2013). Previous studies examining this aspect have typically been conducted along nesting beaches backed by high-density beachfront infrastructure (Kaska et al., 2010; Roe, Clune & Paladino, 2013). By contrast, a distinct buffer of coastal vegetation (100–200 m wide) borders many beaches in our study area. Furthermore, no nesting was recorded during our study on beaches backed by seawalls. These structures modify beach profiles, near-shore bathymetry, and sand exchange (Mosier, 1998; Rizkalla & Savage, 2010), and are often associated with negative impacts on turtle nesting behaviour (Bouchard et al., 1998; Mosier & Witherington, 2002; Miller, Limpus & Godfrey, 2003). Beaches of the northern Sunshine Coast currently have only one short (<200 m) section of seawall, which contrasts with the large engineered seawalls that border many urbanised beaches in other parts of the world (Rizkalla & Savage, 2010).

Variation in wind direction and speed could, hypothetically, also influence nest site selection by turtles by changing surf conditions, particularly the strength and direction of longshore currents, the position of rips, and the number, period and height of breakers across the surf-zones (Lamont & Houser, 2014). Whilst all of these surf-zone properties could be important in determining the longshore position where turtles approaching the beach from the ocean cross the surf-zone, and ultimately crawl onto the beach to nest, there were outside the scope of the study. In fact, these factors have not been measured comprehensively in any other turtle nesting study anywhere.

Our finding of no significant correlation between turtle nest sites and artificial illuminance at the local scale (<100 m) was unexpected, as several studies have identified associations between artificial night light and nesting behaviour of turtles, generally postulating broad hypotheses that turtles may prefer darker beaches, or beach sections, for oviposition (Witherington, 1992; Kamrowski et al., 2012; Mazor et al., 2013). It is theoretically possible that the low influence of illuminance on small-scale nest selection found by us could be the result of nesting that occurred mostly during moonlit conditions. We do, however, consider this unlikely based on the dates when nests were constructed in relation to lunar phases: no nesting was observed during maximum full moon, three nests were laid three days either side of the full moon and one within four days. Furthermore, only two of the five nest sites exposed to artificial light were constructed during this time. The majority (79%) of nests were laid during a new, waxing, or waning moon. We postulate that the most parsimonious hypothesis for our findings is that any light effects on the nesting behaviour of turtles may operate at broader spatial scales than those examined by us (see also Mazor et al., 2013); we stress that this hypothesis remains to be tested, particularly the spatial ambit at which putative light impacts on nesting turtles may occur. Whilst we found no strong evidence for an effect of light on small-scale nest site selection by turtles, artificial night light cannot be excluded to influence turtle nest site selection at regional scales or in settings where contrasts between more brightly lit urban sectors and darker beach sections offer greater ‘contrast’ to make nesting decisions.

Effective turtle conservation is contingent on our ability to identify and manage nesting beaches (and beaches with potential as future nest sites; Hawkes et al., 2009; Fuentes, Limpus & Hamann, 2011; Pike, 2013b). This study outlines a method for completing the first step in this process using recently developed high-accuracy mapping techniques that will be useful in future predictive modelling of nesting habitats. From these models, we describe the environmental features of marine turtle nest sites on the Sunshine Coast and provide values for the most likely ‘envelope’ of preferred nest site conditions. Furthermore, by identifying environmental features that are highly distinct from nest sites, and presumably less suitable as nesting sites, we highlight opportunities where restoration can be conducted to enhance the suitability of beaches for turtle nesting. This information provides a baseline for identifying, predicting, and restoring stretches of the beach and dune scape that are most suitable as turtle nesting sites.

##  Supplemental Information

10.7717/peerj.2770/supp-1 Supplemental Information 1 Spatial distribution of nest sites for 2008–2009 nesting season Spatial distribution of nest sites for 2008–2009 nesting season (Map data ©NearMap Pty. Ltd. 2015).

10.7717/peerj.2770/supp-2 Supplemental Information 2 Spatial distribution of nest sites for the 2009–2010 nesting season Spatial distribution of nest sites for the 2009–2010 nesting season (Map data ©NearMap Pty. Ltd. 2015).

10.7717/peerj.2770/supp-3 Supplemental Information 3 Spatial distribution of nest sites for the 2010–2011 nesting season Spatial distribution of nest sites for the 2010–2011 nesting season (Map data ©NearMap Pty. Ltd. 2015).

10.7717/peerj.2770/supp-4 Supplemental Information 4 Spatial distribution of nest sites for the 2011–2012 nesting season Spatial distribution of nest sites for the 2011–2012 nesting season (Map data ©NearMap Pty. Ltd. 2015).

10.7717/peerj.2770/supp-5 Supplemental Information 5 Spatial distribution of nest sites for the 2012–2013 nesting season Spatial distribution of nest sites for the 2012–2013 nesting season (Map data ©NearMap Pty. Ltd. 2015).

10.7717/peerj.2770/supp-6 Supplemental Information 6 Spatial distribution of nest sites for the 2013–2014 nesting season Spatial distribution of nest sites for the 2013–2014 nesting season (Map data ©NearMap Pty. Ltd. 2015).

10.7717/peerj.2770/supp-7 Supplemental Information 7 Spatial distribution of nest sites for the 2013–2014 nesting season Spatial distribution of nest sites for the 2015–2016 nesting season (Map data ©NearMap Pty. Ltd. 2015).

10.7717/peerj.2770/supp-8 Data S1 Dataset of the nest and reference site environmental attributes

## Additional Information and Declarations

### Data Availability

The following information was supplied regarding data availability: The raw data has been supplied as Data S1.
