# Supplementary figures and images for: Marine turtles are not fussy nesters: a novel test of small-scale nest site selection using structure from motion beach terrain information
Source: PeerJ. 2017 Jan 4;5:e2770. doi: 10.7717/peerj.2770 (PMC5217528; doi:10.7717/peerj.2770)

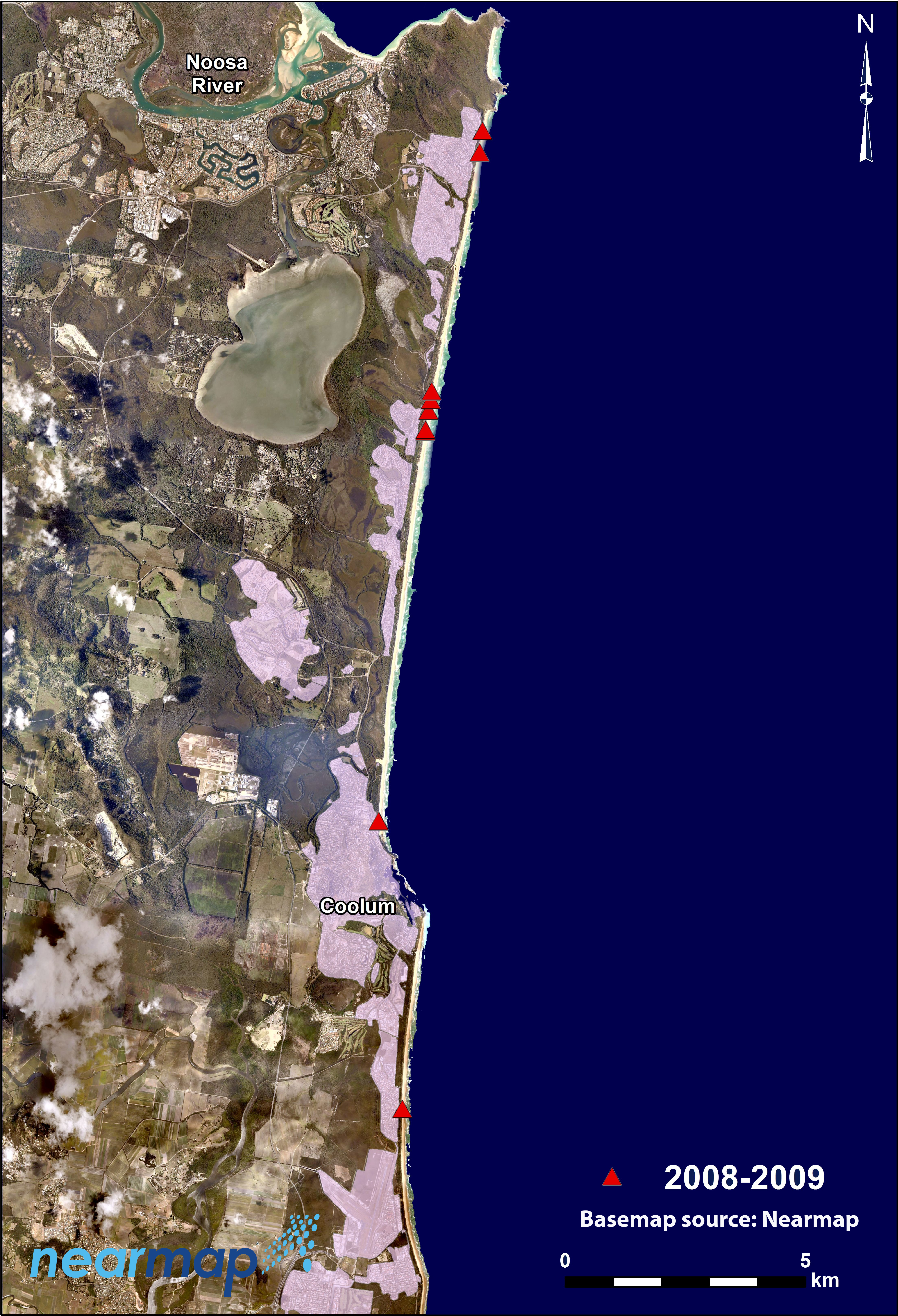

Supplement: Supplemental Information 1 — Spatial distribution of nest sites for 2008–2009 nesting season (Map data ©NearMap Pty. Ltd. 2015). [file peerj-05-2770-s001.png]

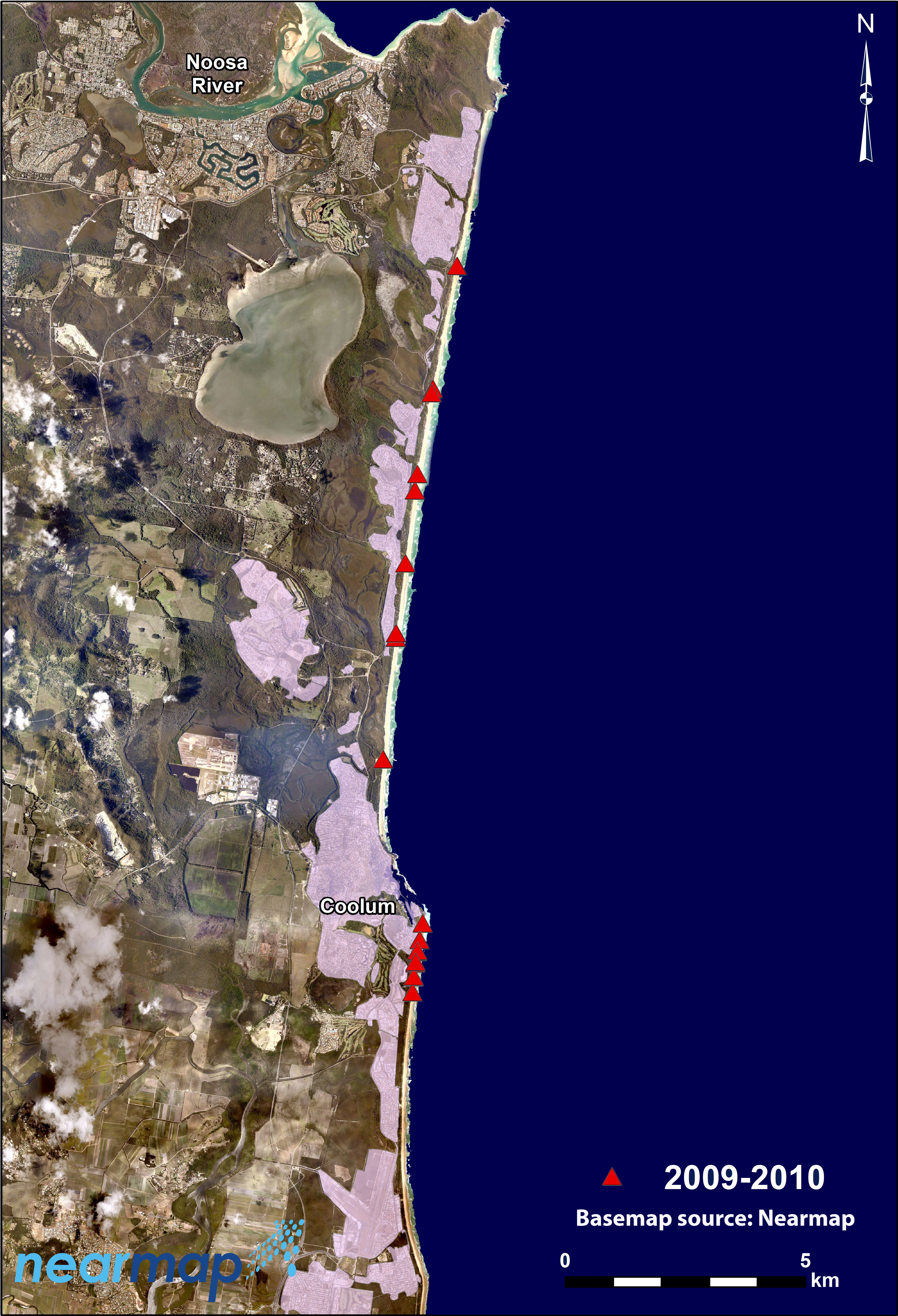

Supplement: Supplemental Information 2 — Spatial distribution of nest sites for the 2009–2010 nesting season (Map data ©NearMap Pty. Ltd. 2015). [file peerj-05-2770-s002.png]

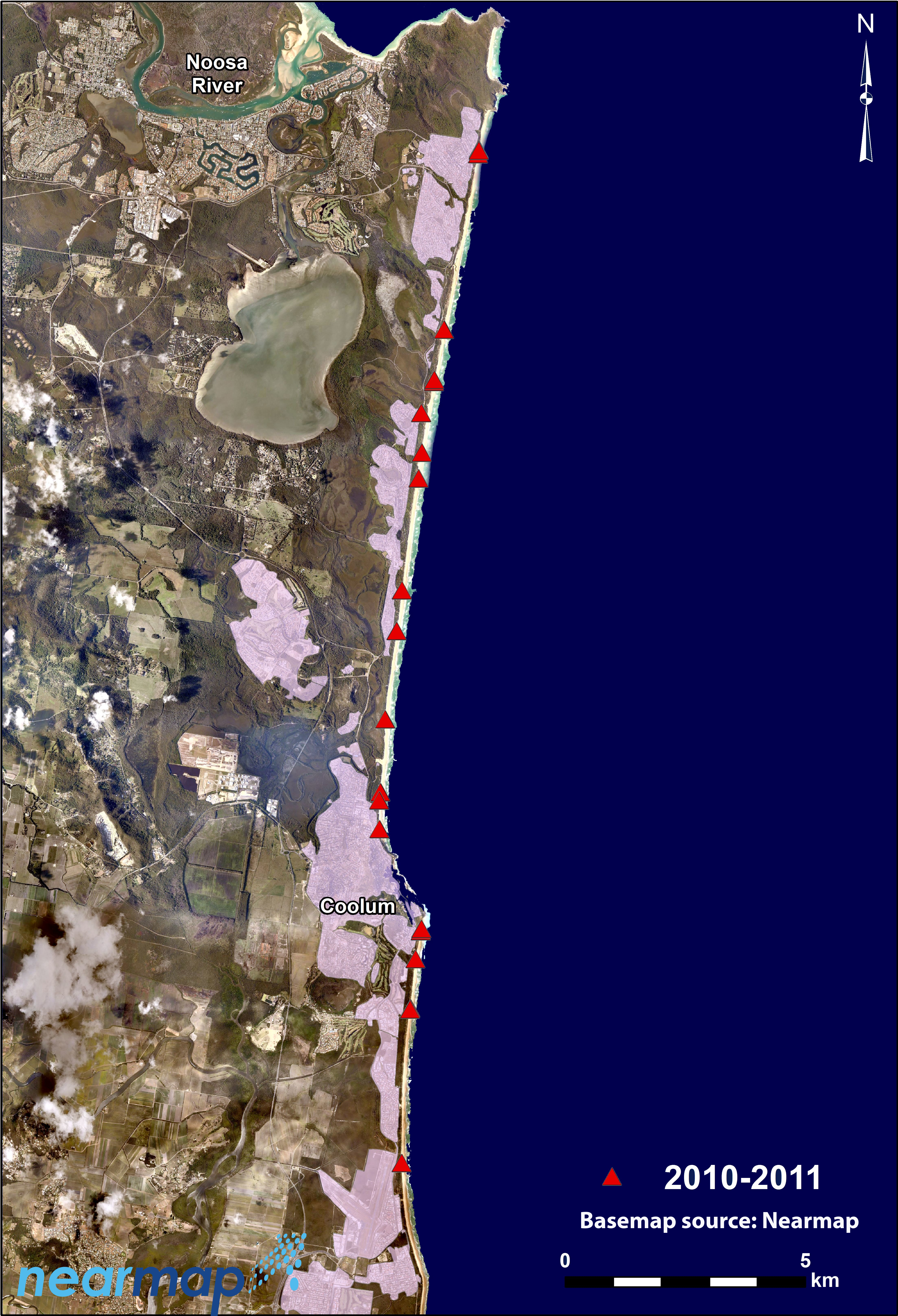

Supplement: Supplemental Information 3 — Spatial distribution of nest sites for the 2010–2011 nesting season (Map data ©NearMap Pty. Ltd. 2015). [file peerj-05-2770-s003.png]

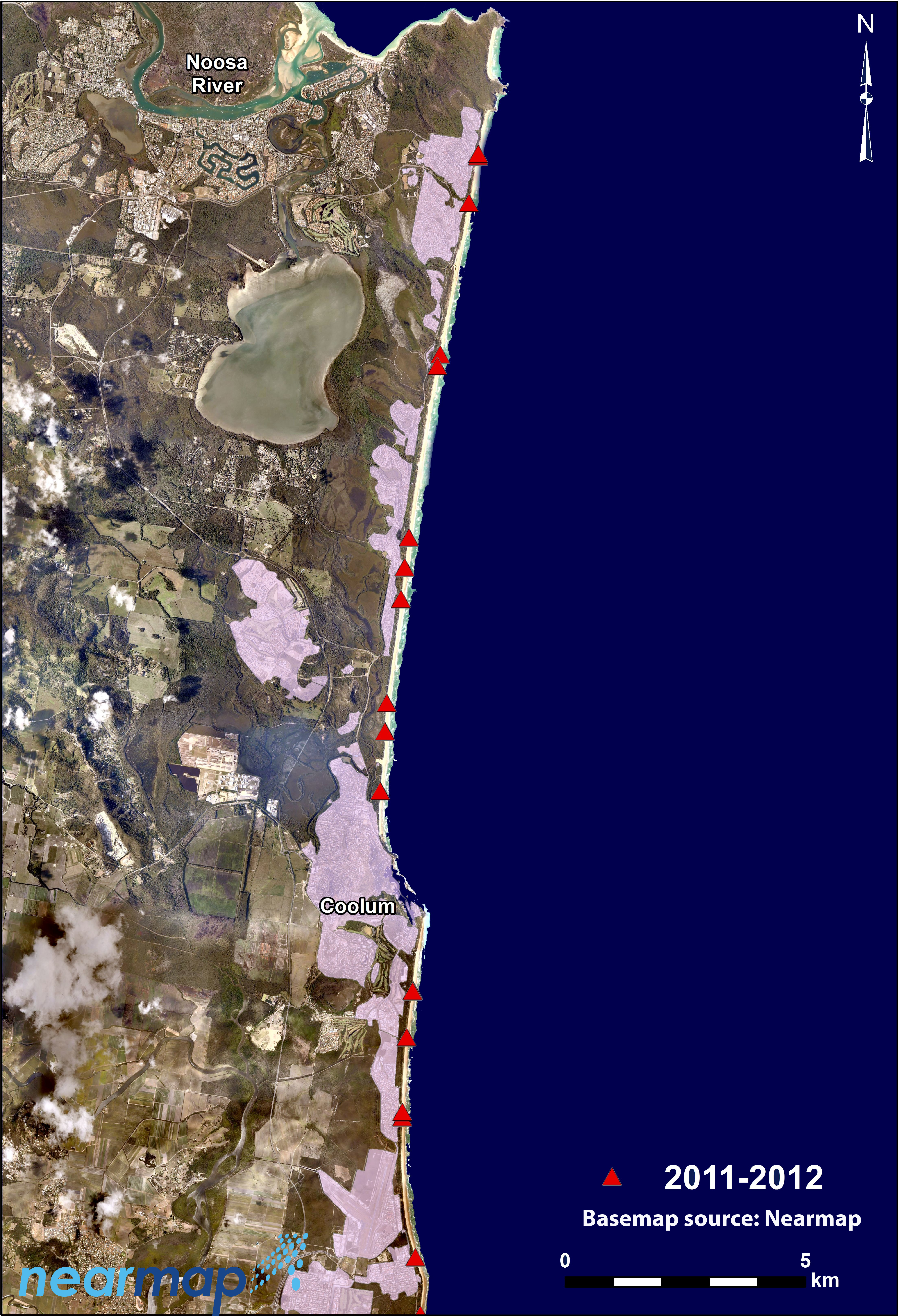

Supplement: Supplemental Information 4 — Spatial distribution of nest sites for the 2011–2012 nesting season (Map data ©NearMap Pty. Ltd. 2015). [file peerj-05-2770-s004.png]

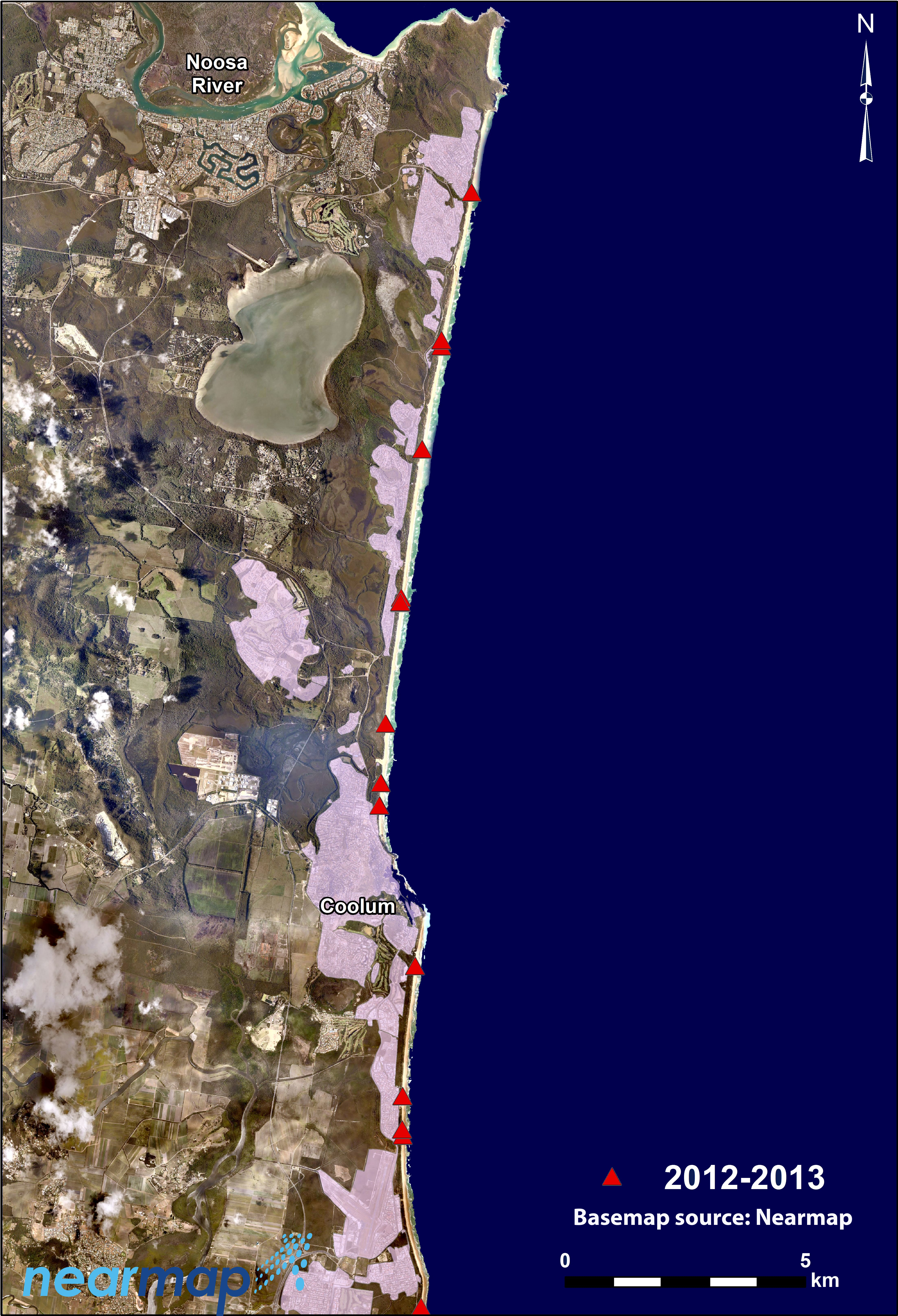

Supplement: Supplemental Information 5 — Spatial distribution of nest sites for the 2012–2013 nesting season (Map data ©NearMap Pty. Ltd. 2015). [file peerj-05-2770-s005.png]

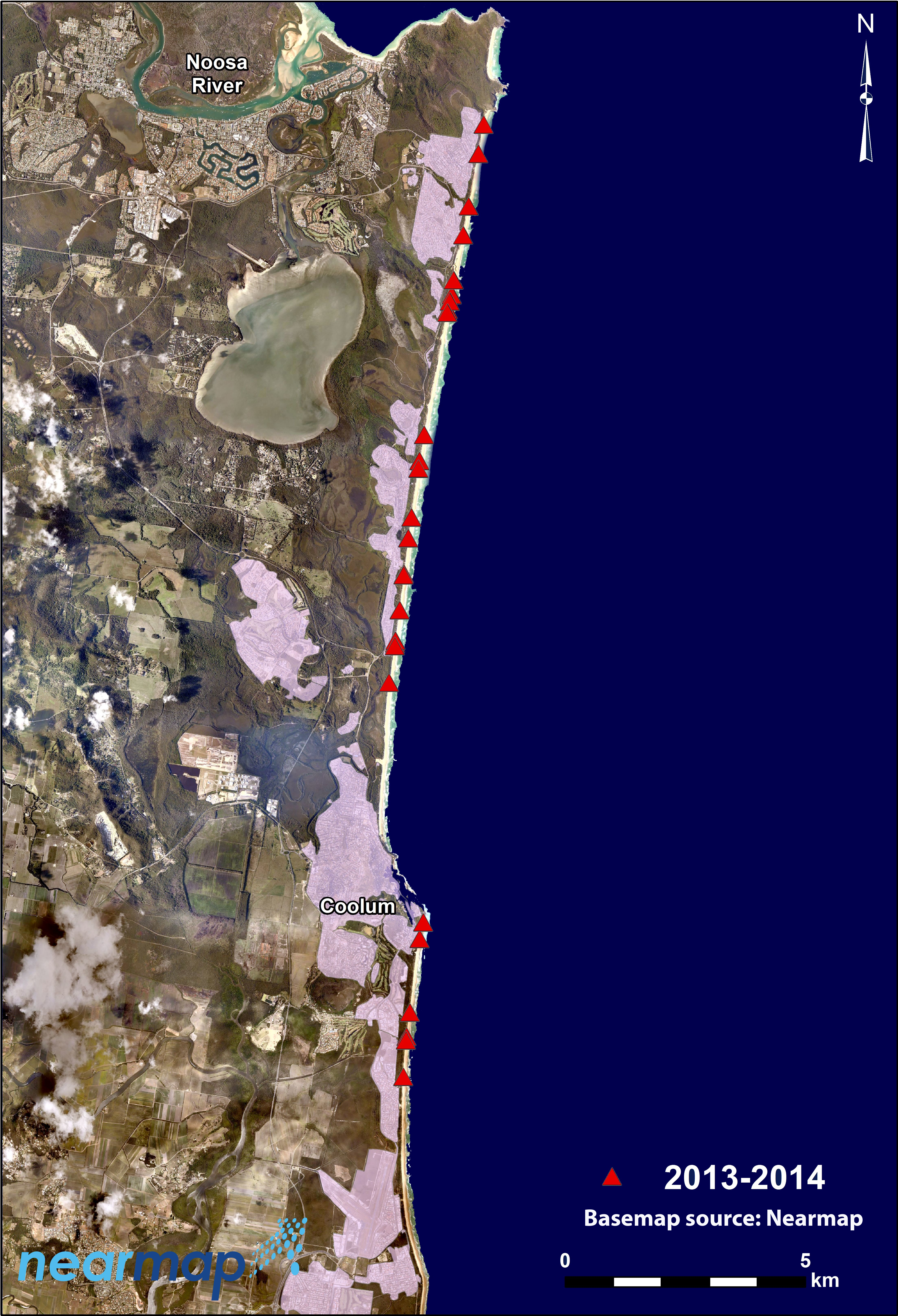

Supplement: Supplemental Information 6 — Spatial distribution of nest sites for the 2013–2014 nesting season (Map data ©NearMap Pty. Ltd. 2015). [file peerj-05-2770-s006.png]

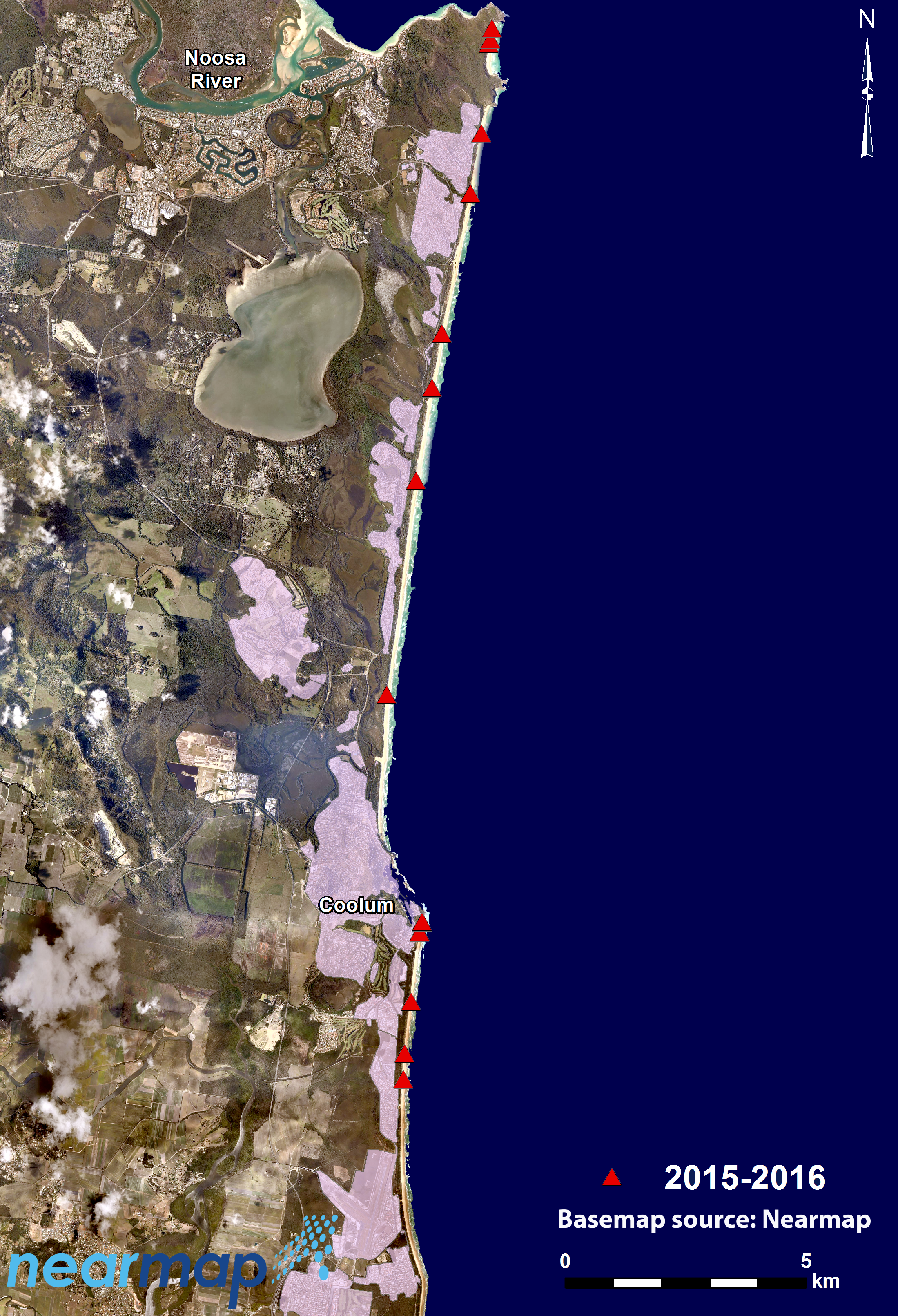

Supplement: Supplemental Information 7 — Spatial distribution of nest sites for the 2015–2016 nesting season (Map data ©NearMap Pty. Ltd. 2015). [file peerj-05-2770-s007.png]
