# Supplementary material for: Marine turtles are not fussy nesters: a novel test of small-scale nest site selection using structure from motion beach terrain information
Source: PeerJ. 2017 Jan 4;5:e2770. doi: 10.7717/peerj.2770 (PMC5217528; doi:10.7717/peerj.2770)
Supplement: Data S1 [file peerj-05-2770-s008.pdf]

ID	NEST / RANDOM	SPECIES	Latitude (deg)	Longitude (deg)	Beach Width (m)	Beach Slope (m)	Dune Width (m)	Dune Slope (deg)	Dune Crest Height (m)	Profile Curvature	Terrain Ruggedness	Dist. to dense Veg. (m)	Dist. to sparse Veg. (m)	Distance to Urban (m)	Illuminance (mcd/m^2)
N1	Nest	Loggerhead	-26.591	153.097	19.514	6.291	6.500	20.782	5.961	-0.072	0.039	4.909	2.460	69.029	1.437
N2	Nest	Loggerhead	-26.415	153.111	17.700	3.211	10.644	3.719	3.283	-0.042	0.018	12.661	1.726	59.641	4.144
N3	Nest	Loggerhead	-26.424	153.108	50.302	3.956	8.501	28.746	10.543	0.016	0.011	12.739	1.562	104.202	2.116
N4	Nest	Green	-26.428	153.108	3.036	9.680	30.971	8.296	7.340	0.013	0.048	2.729	0.985	197.608	9.066
N5	Nest	Loggerhead	-26.430	153.108	11.671	5.592	7.877	18.098	5.698	0.003	0.102	3.162	1.476	179.622	13.722
N6	Nest	Loggerhead	-26.436	153.106	27.979	6.713	16.254	14.635	8.459	-0.084	0.041	2.600	2.000	113.071	8.579
N7	Nest	Loggerhead	-26.449	153.104	32.423	3.488	17.043	13.266	6.820	0.009	0.012	1.789	0.000	355.035	8.193
N8	Nest	Loggerhead	-26.447	153.104	18.251	6.512	13.813	14.635	7.166	0.006	0.015	3.195	0.500	315.087	6.215
N9	Nest	Green	-26.475	153.099	33.743	2.285	15.508	14.621	6.582	-0.001	0.006	6.888	1.200	156.083	4.339
N10	Nest	Loggerhead	-26.495	153.096	14.009	5.627	6.450	18.673	5.377	-0.003	0.026	1.044	0.300	85.866	4.544
N11	Nest	Loggerhead	-26.512	153.094	14.128	-0.665	6.008	25.748	4.455	-0.037	0.005	3.517	0.900	568.177	0.639
N12	Nest	Loggerhead	-26.559	153.100	17.631	3.594	12.281	18.084	7.661	0.002	0.005	8.078	1.868	139.918	1.877
N13	Nest	Loggerhead	-26.563	153.099	22.958	4.677	7.737	18.527	6.140	-0.002	0.030	4.837	0.800	136.781	1.635
N14	Nest	Loggerhead	-26.566	153.099	12.287	6.069	7.009	19.187	5.417	0.008	0.025	8.235	3.523	141.152	1.680
N15	Nest	Loggerhead	-26.569	153.099	10.830	7.064	22.642	7.541	6.335	0.027	0.009	10.296	0.600	194.833	0.257
N16	Nest	Loggerhead	-26.552	153.101	21.257	0.678	2.571	19.909	6.966	-0.036	0.033	2.642	1.800	56.294	0.392
N17	Nest	Loggerhead	-26.597	153.098	20.348	7.172	9.330	10.692	4.831	-0.005	0.013	6.629	2.915	202.988	3.479
N18	Nest	Loggerhead	-26.433	153.107	17.424	5.995	14.360	16.071	7.934	-0.010	0.009	10.878	1.345	165.940	7.070
N19	Nest	Loggerhead	-26.560	153.100	2.000	10.154	15.926	8.124	5.126	0.014	0.054	9.513	1.063	151.076	1.635
R1	Random	Loggerhead	-26.609	153.100	7.376	3.031	11.800	12.756	5.523	-0.004	0.043	0.943	0.000	55.145	4.802
R2	Random	Loggerhead	-26.609	153.100	9.067	3.599	10.119	12.480	4.983	-0.009	0.008	6.100	0.141	58.000	2.363
R3	Random	Loggerhead	-26.507	153.094	33.230	4.781	4.545	30.418	6.773	0.004	0.001	13.447	7.849	588.782	1.635
R4	Random	Loggerhead	-26.507	153.094	33.725	5.163	2.791	24.696	5.645	-0.001	0.001	20.577	15.369	597.234	1.561
R5	Random	Loggerhead	-26.560	153.100	6.708	9.260	15.940	9.625	5.620	-0.324	0.167	0.000	7.940	128.763	1.450
R6	Random	Loggerhead	-26.560	153.100	8.322	5.228	6.259	12.653	3.992	-0.026	0.011	6.908	0.000	150.030	1.620
R7	Random	Loggerhead	-26.433	153.107	18.515	6.031	12.637	17.726	7.990	-0.009	0.016	17.395	0.283	174.614	4.937
R8	Random	Loggerhead	-26.433	153.107	21.648	5.285	16.920	13.674	7.722	-0.002	0.021	10.653	0.566	161.208	7.611
R9	Random	Green	-26.597	153.098	20.756	6.824	8.200	15.806	5.733	0.003	0.013	7.806	1.414	202.556	4.300
R10	Random	Green	-26.597	153.098	18.604	6.443	7.432	18.231	5.993	-0.005	0.012	14.290	0.283	208.866	3.322
R11	Random	Loggerhead	-26.413	153.111	16.931	2.227	13.823	3.049	4.088	-0.015	0.024	0.000	1.900	99.081	1.263
R12	Random	Loggerhead	-26.413	153.111	2.200	0.108	38.869	0.626	1.797	0.007	0.005	8.981	1.860	128.191	5.314
R13	Random	Loggerhead	-26.552	153.101	12.947	6.757	7.300	12.954	6.101	-0.009	0.018	2.003	0.100	65.734	1.080
R14	Random	Loggerhead	-26.552	153.101	12.728	6.938	6.657	14.617	6.163	-0.004	0.007	15.533	12.667	79.555	0.714
R15	Random	Loggerhead	-26.491	153.097	27.922	4.525	9.818	15.119	6.056	0.002	0.005	7.497	1.342	91.351	4.802
R16	Random	Loggerhead	-26.491	153.097	29.603	4.312	8.386	18.505	6.158	0.007	0.132	0.000	1.400	73.682	2.363
R17	Random	Loggerhead	-26.568	153.099	12.700	7.284	21.557	7.456	6.387	-0.027	0.036	1.780	0.000	184.532	1.251
R18	Random	Loggerhead	-26.569	153.099	14.080	6.127	20.841	10.602	7.088	-0.012	0.006	16.510	0.200	202.200	1.121
R19	Random	Loggerhead	-26.566	153.099	11.610	5.874	7.580	18.352	5.347	0.006	0.035	0.200	0.000	131.856	1.826
R20	Random	Loggerhead	-26.566	153.099	6.552	6.253	10.211	14.123	5.108	-0.009	0.019	7.224	1.200	142.144	1.195
R21	Random	Green	-26.563	153.099	22.840	4.189	8.013	19.804	6.455	-0.007	0.016	7.543	0.300	141.028	1.760
R22	Random	Green	-26.564	153.099	22.830	4.611	7.653	19.044	6.162	-0.010	0.002	10.435	1.697	143.014	1.195
R23	Random	Loggerhead	-26.559	153.100	18.840	5.862	8.538	15.805	7.076	-0.004	0.041	4.684	0.583	132.639	2.407
R24	Random	Loggerhead	-26.559	153.100	13.262	5.021	12.900	16.608	7.600	-0.069	0.107	0.000	0.894	121.696	2.639
R25	Random	Loggerhead	-26.552	153.101	12.417	6.958	4.318	27.279	6.672	0.009	0.088	0.000	0.700	61.814	0.890
R26	Random	Loggerhead	-26.552	153.101	5.600	9.864	14.410	10.285	6.814	0.002	0.018	6.512	3.513	74.953	1.323
R27	Random	Loggerhead	-26.512	153.094	14.406	-1.378	5.700	28.955	4.544	0.111	0.104	0.000	1.500	562.100	0.923
R28	Random	Loggerhead	-26.512	153.094	24.910	-1.751	4.904	37.949	5.254	-0.004	0.211	1.020	0.200	551.514	1.229
R29	Random	Loggerhead	-26.495	153.096	12.462	5.639	6.239	14.833	5.032	-0.023	0.003	9.000	0.906	90.471	5.881
R30	Random	Loggerhead	-26.495	153.096	14.547	5.183	4.789	22.632	5.323	0.026	0.039	0.000	0.100	78.339	3.086
R31	Random	Loggerhead	-26.475	153.099	33.280	2.257	14.374	14.633	6.246	0.065	0.113	0.447	0.000	147.580	4.379
R32	Random	Loggerhead	-26.475	153.099	32.733	2.735	16.907	15.541	7.308	0.003	0.001	33.121	3.008	187.171	7.752
R33	Random	Loggerhead	-26.447	153.104	19.969	5.276	13.240	15.902	7.154	0.000	0.014	0.000	0.400	298.277	3.850
R34	Random	Loggerhead	-26.447	153.104	16.668	6.961	14.641	12.827	6.983	0.024	0.037	1.404	0.000	315.626	5.217
R35	Random	Loggerhead	-26.449	153.104	32.247	2.130	14.684	15.980	6.643	-0.005	0.030	0.000	0.200	336.305	3.262
R36	Random	Loggerhead	-26.449	153.104	32.976	2.953	14.618	16.559	7.011	0.000	0.001	21.992	4.826	346.733	7.202
R37	Random	Loggerhead	-26.442	153.105	23.444	5.995	10.984	16.821	6.827	-0.010	0.023	9.936	2.470	157.867	4.586
R38	Random	Loggerhead	-26.442	153.105	14.100	6.100	14.727	17.284	7.082	-0.011	0.136	6.720	0.500	136.312	8.819
R39	Random	Loggerhead	-26.436	153.107	18.668	4.663	19.007	14.222	6.897	0.000	0.004	10.102	4.305	142.004	5.881
R40	Random	Loggerhead	-26.436	153.107	31.419	6.070	13.300	17.661	8.574	0.020	0.006	15.446	12.264	142.352	6.101
R41	Random	Loggerhead	-26.430	153.108	11.957	5.671	8.819	19.274	6.254	0.027	0.007	1.105	0.000	191.951	9.581
R42	Random	Loggerhead	-26.430	153.108	12.926	4.236	11.510	14.725	5.614	0.048	0.031	5.914	0.000	185.311	6.101
R43	Random	Loggerhead	-26.428	153.108	15.111	5.465	18.800	11.234	7.759	0.004	0.068	2.631	0.000	196.621	6.752
R44	Random	Loggerhead	-26.428	153.108	1.900	7.566	32.047	7.794	6.963	0.020	0.056	1.700	0.000	192.844	8.268
R45	Random	Loggerhead	-26.424	153.109	50.525	3.651	8.485	16.592	8.307	-0.020	0.028	0.000	2.000	89.889	4.714
R46	Random	Loggerhead	-26.424	153.108	52.200	4.080	7.669	19.057	8.268	-0.024	0.001	17.147	6.301	119.407	5.314
R48	Random	Loggerhead	-26.415	153.111	32.163	5.376	9.646	1.399	3.678	-0.013	0.010	0.000	0.860	50.990	4.502
R49	Random	Loggerhead	-26.591	153.098	20.169	6.828	8.010	18.925	6.074	-0.006	0.004	24.664	17.018	89.275	1.635
R50	Random	Loggerhead	-26.591	153.098	23.244	5.684	8.996	15.976	5.684	0.001	0.006	20.957	10.382	82.607	2.256
R51	Random	Loggerhead	-26.585	153.098	17.560	5.653	7.600	17.169	5.925	0.017	0.468	0.000	6.251	62.000	1.929
R52	Random	Loggerhead	-26.585	153.097	16.889	4.928	7.900	19.032	6.051	0.004	0.001	15.231	6.155	90.449	3.144
